# Modeling Blood–Brain Barrier Permeability to Solutes and Drugs In Vivo

**DOI:** 10.3390/pharmaceutics14081696

**Published:** 2022-08-15

**Authors:** Ulrich Bickel

**Affiliations:** 1 Department of Pharmaceutical Sciences, Jerry H. Hodge School of Pharmacy, Texas Tech University Health Sciences Center, Amarillo, TX 79106, USA; ulrich.bickel@ttuhsc.edu; Tel.: +1-806-414-9236; Fax: +1-806-356-4034; 2 Center for Blood-Brain Barrier Research, Jerry H. Hodge School of Pharmacy, Texas Tech University Health Sciences Center, Amarillo, TX 79106, USA

**Keywords:** pharmacokinetics, blood–brain barrier, compartmental models, physiologically based PK models

## Abstract

Our understanding of the pharmacokinetic principles governing the uptake of endogenous substances, xenobiotics, and biologicals across the blood–brain barrier (BBB) has advanced significantly over the past few decades. There is now a spectrum of experimental techniques available in experimental animals and humans which, together with pharmacokinetic models of low to high complexity, can be applied to describe the transport processes at the BBB of low molecular weight agents and macromolecules. This review provides an overview of the models in current use, from initial rate uptake studies over compartmental models to physiologically based models and points out the advantages and shortcomings associated with the different methods. A comprehensive pharmacokinetic profile of a compound with respect to brain exposure requires the knowledge of BBB uptake clearance, intra-brain distribution, and extent of equilibration across the BBB. The application of proper pharmacokinetic analysis and suitable models is a requirement not only in the drug development process, but in all of the studies where the brain uptake of drugs or markers is used to make statements about the function or integrity of the BBB.

## 1. Introduction

**Figure 1:**
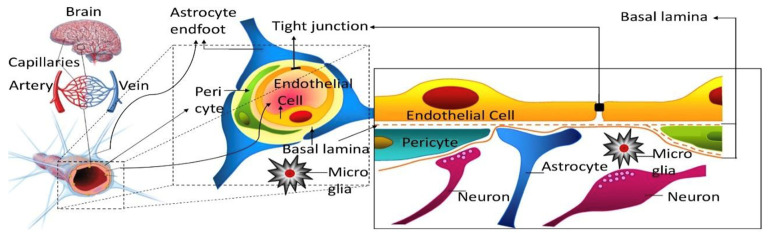
Scheme of the BBB: The endothelial cells of the capillaries are connected by tight junctions and form a physical and biochemical barrier. The pericytes and astrocytes play critical roles in the induction and maintenance of the endothelial barrier properties. Microglial cells and neurons also secrete signals, which can influence the endothelial cells. The diameter of the capillaries is on the order of 7–10 µm. Figure adapted from Reference [3] with permission. Copyright 2010, Elsevier.

It has been over 120 years since the earliest publications on the distinct features of the brain vasculature compared to other organs, which over time gave rise to the concept of a “blood–brain barrier” (BBB). As recently reviewed [1], there have been many misconceptions along the way about its structure and function. It is now established that the physical and biochemical BBB is formed by the endothelial cells of the brain microvasculature, which are connected by tight junctions (Figure 1). The barrier function is induced or modulated by other elements of the “neurovascular unit” [2], including pericytes, astrocytes, neurons, and microglial cells. Some misunderstandings linger to this day in parts of the literature. One area concerns the methods of measuring the brain uptake of drugs and the proper evaluation and interpretation. The present review will provide an overview of the development and application of modeling approaches to describe the pharmacokinetics (PK) of brain uptake of low molecular weight drug-like solutes and macromolecules. We will not attempt to include all of the aspects of the extensive and rapidly growing literature in this field, but aim to focus on practical applicability with some examples.

The purpose of the PK models covers a range from the evaluation and the fitting of sets of experimental data that are as good as possible to simulations and predictions based on few or no experimental data, to employing complex physiologically based pharmacokinetic (PBPK) models with parameter values obtained from in silico, in vitro, and in vivo studies. Which model is appropriate also depends on the experimental design in each case and the physicochemical characteristics of the agent to be evaluated. Macromolecules differ greatly in kinetic behavior from small molecule drugs and studying poorly permeable substances requires a different experimental design and PK evaluation approaches than highly permeable drugs. From an experimental perspective, it is feasible in preclinical studies to obtain blood and (brain) tissue samples at multiple time points, while clinical studies are typically limited to blood sampling, and occasionally CSF sampling, and noninvasive imaging modalities (MRI, SPECT, PET), if applicable. Finally, a distinction can be made, based on whether the parameter of primary interest is the uptake rate across the BBB or the brain exposure (fraction of a dose, or extent). These models will be presented, from the simple to the more complex.

## 2. Models Assuming Unidirectional Brain Uptake

As the name implies, unidirectional organ uptake models are based on the concept that for the duration of the experiment, a drug or tracer diffuses (or is undergoing transport) only from the blood side (luminal) to the brain side (abluminal) across the BBB. Provided that there is no significant backflux, it allows the determination of the initial rate of brain uptake. The approach can be most readily illustrated considering the examples of carotid injection and carotid artery brain perfusion techniques.

### 2.1. Brain Uptake Index (BUI)

The technique, as introduced by Oldendorf [4,5], uses radiotracers labeled with different isotopes and measures the brain tissue-extraction ratio (brain concentration: injectate concentration) of a compound during a single pass through the cerebral vasculature after bolus injection into the carotid artery under anesthesia. BUI (as a percentage) is then estimated from the brain extraction ratio of the test substance (*E_test_*), relative to that of a permeable reference substance (*E_refP_*) with a known, near total extraction:

(1) $$BUI=\frac{E_{test}}{E_{refP}}\times 100=\frac{C_{test}in\;brain/C_{test}in\;injectate\;}{\;\;\;\;C_{refP}in\;brain/C_{refP}in\;injectate}\times 100$$

where *C_test_* refers to the concentrations of the test substance and *C_refP_* refers to the concentrations of the permeable reference. The examples of highly permeable substances with a complete (100%) extraction include iodoamphetamine [6] and diazepam [7]. In order to correct for brain intravascular volume, an additional reference substance was introduced, which is assumed to show no tissue uptake during the single passage.

*BUI* in percent is then calculated as the ratio:

(2) $$BUI=100\times \left(E_{test}-E_{refV}\right)/E_{refP}$$

where *E_refV_* is the apparent “extraction” of the vascular marker (non-permeant reference). *E_test_* can then be used to calculate the permeability surface area product (*PS*), applying a rearranged Renkin–Crone equation:

(3) PS=−F ln(1−E)

where *F* is the tissue perfusion flow rate. The BUI method allows wide flexibility in the composition of the injectate and the concentrations of the test substance. In the BUI technique, the brain tissue is treated as one compartment. It has been successfully applied to study, in particular, the BBB transport of nutrients, including the characterization of saturable transport by applying Michaelis–Menten kinetics [8]. A limitation of the technique is its low sensitivity for poorly permeable solutes. For example, the brain uptake of the classical CNS-active drug, morphine could not be distinguished from [^14^C]sucrose, which behaves as a vascular marker in a single-pass experiment [9].

### 2.2. In Situ Brain Perfusion

These techniques perfuse the brain in anesthetized animals with oxygenated buffer solutions via the carotid artery. Similar methods have been established in the rat [7], guinea pig [10], and mouse [11,12] and are in widespread use. The pharmacokinetic principle is analogous to the BUI method, in the assumption that the initial rate of brain uptake can be measured as long as the effective tissue concentrations are much lower than the perfusate concentration and unidirectional transport prevails. The evaluation is based on a plot of the apparent volume of distribution (*V_D_*) in the brain compartment against perfusion time (*T*), where *V_D_* is the ratio of the amount of the test substance in the brain per unit weight, *A_br_* (e.g., nmol/g brain) over the perfusate concentration (*C_perf_*) of the analyte (e.g., nmol/mL), with *C_perf_* being constant. With a unidirectional uptake, this results in a linear increase, and the slope of a linear regression line represents the unidirectional transfer constant, *K_in_*, from the perfusate fluid into the brain compartment, which is a clearance parameter, e.g., in units of [mL/min]:

(4) $$V_{D}=\frac{A_{br}}{C_{perf}}=K_{in}T+V_{i}$$

The y-intercept of the regression line represents an “initial” volume of distribution, *V_i_*. It corresponds to the sum of the intravascular volume, *V*_0_, which in rodents typically is in a range of 8–10 µL/g [13], and a potential residual volume, which is physiologically difficult to characterize, e.g., caused by nonspecific binding to the vascular wall, or associated with cellular components of the BBB. In cases where substantially higher values of *V_i_* are observed, the inclusion of a marker substance, such as albumin or inulin, which is expected to undergo negligible transport across the BBB, can be applied to experimentally measure the intravascular space. *K_in_* approaches the value of the PS product at the BBB within 10% for conditions of permeability-limited transport (low E), when flow F is greater than 5x PS. Depending on the test compound, in situ perfusions are typically conducted over a few seconds up to several minutes. Similar to BUI, the experimental advantages are that the perfusate compositions and concentrations of the test substance can be controlled within wide limits, outside of the conditions tolerated in the whole animal with systemic administration. On the other hand, the sensitivity for accurate permeability measurements of analytes with low permeability is superior to the single-pass technique, but is still limited. The extended perfusion times (e.g., 10–20 min) require the inclusion of oxygen carriers, such as washed erythrocytes in the perfusate, to avoid hypoxic conditions [10].

### 2.3. Intravenous Injection

The most physiological technique for the analysis of BBB transport is the intravenous administration (as a bolus or infusion), followed by the analysis of the concentration time course in blood and brain. To obtain reliable and correct measurements, several conditions have to be met in such studies. First, considering the free drug hypothesis [14], the free fraction in plasma needs to be determined by microdialysis in vivo, or by ex vivo techniques, such as ultrafiltration. This will be discussed in more detail below in Section 4. Second, if metabolism occurs, the analytical method used must be able to measure the intact substance in the plasma and tissue.

#### 2.3.1. Multiple Time Point Analysis

Depending on the experimental design, different options for the pharmacokinetic evaluation of brain uptake are available. With a series of brain and plasma samples taken at different time points, the multiple time-point graphical evaluation, also known as a Patlak plot, is frequently used [15,16,17]. In its original form, it modeled the brain tissue as consisting of several reversible compartments and one irreversible compartment [17]. A practical example is the intracellular entrapment of tracers, such as deoxy-glucose or α-amino-isobutyric acid. Under the condition that the reversible compartments are in rapid exchange with the plasma compartment, Patlak and colleagues showed that the unidirectional brain uptake can be analyzed by plotting the time-dependent apparent volume of distribution against the ratio of the plasma area under the curve (AUC) from time zero to time T and terminal plasma concentration at time T:

(5) $$\frac{A_{br}\left(T\right)}{C_{p}\left(T\right)}=K_{in}\frac{\int_{0}^{T}C_{p}\left(t\right)dt}{C_{p}\left(T\right)}+V_{i}$$

where *Cp(T)* is the plasma concentration at sampling time *T*; and *V_i_* is the initial volume of distribution, as introduced above. The term [*AUC*_0_*^T^*/*C_p_*(*T*)] gives a value in units of time. It is, however, distinct from the experimental time in all of the cases where plasma concentrations are not constant, and is labeled “effective time” or “stretch time”. After the initial phase of rapid equilibration of *V_i_*, a phase of linear increase in the plotted data is expected, as long as the unidirectional uptake into the brain compartment occurs. This linear phase allows for an analysis by linear regression, yielding *K_in_* as the slope of the regression line as described above for the in situ perfusion technique. The intravascular content may be experimentally accounted for by the inclusion of a vascular marker injected shortly before the terminal experimental time and tissue sampling, or by a vascular wash procedure to clear out the intravascular content from the tissue. Both options, if appropriately applied, yield equivalent corrections, as recently demonstrated [13].

#### 2.3.2. Single Time Point Analysis

This evaluation only requires one terminal brain sample and sufficient blood samples obtained over the experimental period to accurately describe the plasma concentration time-course of the test substance. The calculation of the brain uptake clearance is then performed, utilizing a version of Equation (5) re-arranged as follows:

(6) $$K_{in}=\frac{\left(V_{D}-V_{0}\right)C_{p}\left(t\right)}{AUC_{0}^{t}}$$

where *V_D_* corresponds to the apparent brain volume of distribution (*A_br_*/*C_pT_*); and *V*_0_ is the intravascular volume. The latter needs to be either measured experimentally, using a vascular marker, taken from the literature, or eliminated by a buffer wash through the left heart ventricle. When serial blood samples can be taken from one animal, the single time-point method has the advantage of reducing the number of experimental animals. The disadvantage is that it is challenging to select the best terminal sampling time, up to which the unidirectional BBB transport can be expected. There is a trade-off between sensitivity for the analysis of low permeability substances in the brain tissue and the risk of violating the initial rate condition (see also the next section). At short experimental times, when the tissue concentrations beyond the BBB are relatively low and the intravascular concentrations are still high, minor errors in correction for vascular content may have outsized effects on the apparent *K_in_*.

### 2.4. Caveats Associated with Unidirectional Uptake Models

**Figure 2:**
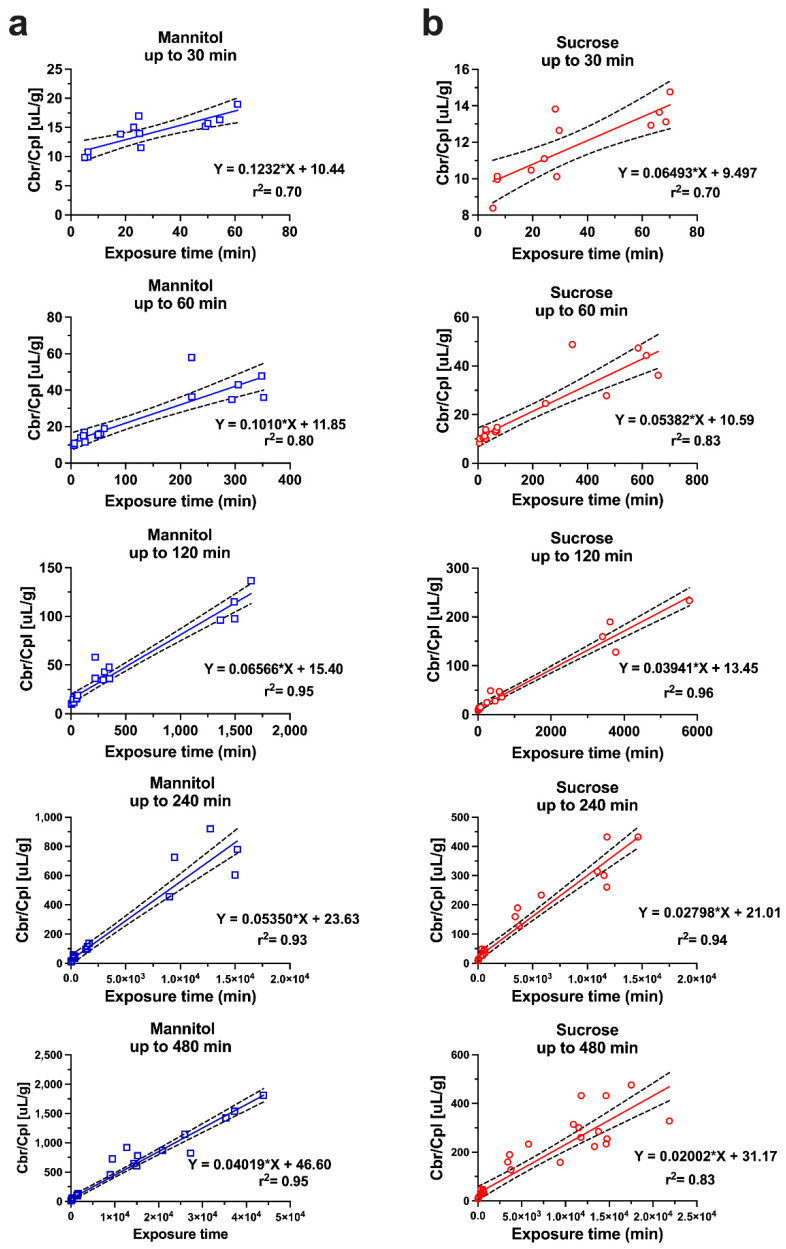
Multiple time point-analysis covering different sampling time points up to 480 min for (**a**) mannitol and (**b**) sucrose. Dashed lines represent the 95% confidence intervals. *n* = 4–7 animals per marker per time point. Reprinted with permission from Reference [19]. Copyright 2022, Springer Nature.

**Figure 3:**
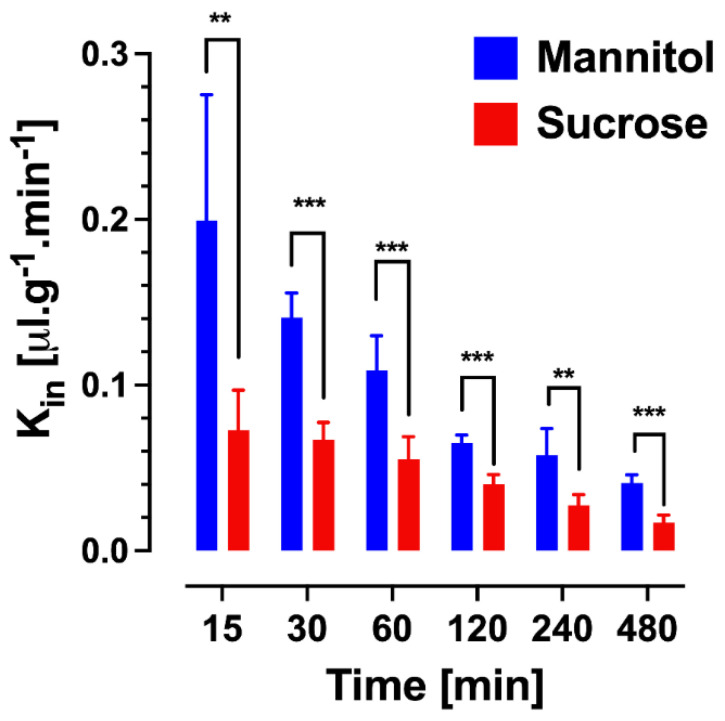
Single time point analysis of sucrose and mannitol brain uptake clearance values (K_in_) at different time points from 15 to 480 min (*n* = 4–7 animals per marker per time point). Sucrose and mannitol values at each time point were compared by *t*-test. ** *p* < 0.01, *** *p* < 0.001. Reprinted with permission from Reference [19]. Copyright 2022, Springer Nature.

While the original Patlak model was developed assuming an irreversible brain compartment, the multiple-time graphical method has found widespread application in the analysis of the uptake of compounds, which are unbound or reversibly bound by brain tissue beyond the BBB. In these scenarios, a deviation from the linearity at later time points, when the backflux cannot be neglected, is theoretically expected. Applying a linear regression analysis should then result in a systematic underestimation of the apparent value of K_in_ and an overestimation of *V_i_*. Unfortunately, it is difficult to identify such errors in practice from the output of linear regression of the experimental data, which are also inherently associated with error. The dilemma has been explored with the example of an in situ brain perfusion study of [^14^C]iodoacetamide, a moderately lipophilic test substance [18]. The data acquired experimentally, up to 40 s perfusion time, were corrected for vascular content by [^3^H]inulin. Within the experimental period, the [^14^C]iodoacetamide reached an apparent V_D_ in brain of 0.15–0.2 mL/g, indicating quite rapid BBB transport. A linear regression of the Patlak plot (after vascular correction) gave a value of 4.39 ± 0.33 × 10^−3^ mL/s/g and a V_i_ not significantly different from zero, as predicted. The same data were fitted to a two-compartment model, allowing for backflux from the brain, resulting in a slightly higher *K_in_* of 5.08 ± 0.14 × 10^−3^ mL/s/g. When the parameters of the two-compartment model were applied to simulate the later data points from 60 up to 240 s, the Patlak analysis still showed an r^2^ coefficient of 0.973 in support of linearity. However, the K_in_ estimate dropped more than half to 2.06 ± 0.24 × 10^−3^ mL/s/g and the *V_i_* increased to 0.15 mL/g. Therefore, the assumption of linearity based on the r^2^ values close to one cannot be taken as proof of an accurate estimate of K_in_. Importantly, this conclusion not only applies to the compounds with moderate or high permeability, but also to poorly permeable substances, such as the hydrophilic markers [^13^C]sucrose and [^13^C]mannitol. A recent study was performed with IV bolus injections of these compounds in awake mice, followed by the sampling of blood and brains, with terminal sampling time points between 30 min and 480 min [19]. Separate Patlak analyses were conducted, using multiple-time point graphical analysis for data covering experimental periods either up to 30, 60, 120, 240, or 480 min (Figure 2). Analogous to the simulation scenario with [^14^C]iodoacetamide, the inclusion of the late experimental time points resulted in a substantial underestimation of K_in_, with values for both mannitol and sucrose decreasing at 120 min by 40–50% and by 480 min around 70% from the estimates obtained, with 30 min as the terminal sampling time. When the same dataset was analyzed using the single time technique, there was a similar gradual decline in the apparent *K_in_* calculated from the late terminal time points of brain tissue sampling (Figure 3), while the mannitol and sucrose values compared at each time point remained significantly different. The figure also reveals that the variability coefficient of the *K_in_* estimates at the earliest time point (15 min) is highest, likely due to the argument outlined above, about the impact of the intravascular content.

### 2.5. PK Analysis in Brain Imaging Techniques

Notwithstanding the limitations of the standard version of the Patlak analysis, the graphical evaluation technique is widely used in the fields of PET imaging and MRI, where a time series can be acquired of the regional brain uptake and from the regions over large arteries, as reference measurements of the input function. Patlak and Blasberg presented a generalized, non-linear version of their original approach, which allowed for the loss of the test substance from the brain [16]. The modified equation:

(7) $$\frac{A_{br}\left(T\right)}{C_{p}\left(T\right)}=K_{in}\frac{\int_{0}^{T}e^{-k_{b}\left(T-t\right)}\;C_{p}\left(t\right)dt}{C_{p}\left(T\right)}+\left(fV_{e}+V_{p}\right)$$

includes a rate constant *k_b_* for the test substance leaving the brain tissue, where it is assumed that *k_b_* << *K_in_*. The term *fV_e_* denotes a fraction of the extravascular distribution volume in brain tissue, and *V_p_* is brain plasma space. The non-linear generalized Patlak equation is used in the evaluation of preclinical and clinical PET and MRI data [20,21,22]. In addition, for the PET imaging of radioligands showing reversible binding to receptors or enzymes in the brain, Logan et al. proposed a graphical analysis to estimate the steady state volume of the distribution of a tracer from the slope of a linear segment of the plot [23]. Subsequently, a number of variations of the “Logan plot” were introduced in an effort to reduce the bias for the underestimation of V_D_, caused by noisy data in the original version [24]. Due to their non-invasiveness, advanced imaging modalities such as dynamic-contrast enhanced MRI (DCE-MRI) and fluid-attenuated inversion recovery MRI (FLAIR MRI) are in widespread clinical use. The regional BBB dysfunctions can be detected and quantified in multiple disease states, ranging from neuroinflammatory diseases, such as multiple sclerosis [25] and brain tumors [26], to ischemic brain diseases [27]. The enhanced sensitivity for the detection of subtle BBB leakage in patients could recently be shown under high field strength (7T) MRI [28].

## 3. Compartmental Models of Brain Uptake

The methods discussed in the previous sections are based on the presence of at least one central compartment representing the input source for brain uptake, and possibly additional compartments, which can determine the characteristic concentration–time course of a drug in the circulation after systemic administration, and one or more brain compartments. However, the determination of the initial rate of uptake K_in_ from the intravascular space across the BBB does not require analytic or numerical solutions of intercompartmental transfer rates. The plasma AUC in Equations (5) and (6) may be readily calculated by a non-compartmental trapezoidal approach. The unidirectional clearance transfers the analyte from the intravascular fluid into brain tissue, which is reduced to a single compartment, represented by an apparent volume of distribution. However, to analyze the exchange in both directions across the BBB, and the potential transport between additional compartments within the tissues of the central nervous system, compartmental PK models or PKPB models are required.

**Figure 4:**
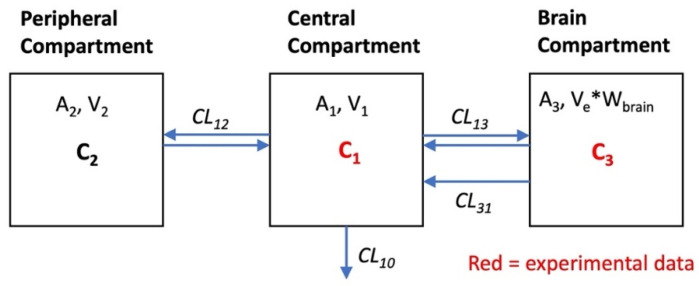
Schematic of a three-compartment semi-physiologic model for the pharmacokinetic study. Parameter definitions are given in the methods above. A_1_, V_1_, C_1_ are the amount, the volume, and the concentration in the central compartment 1, analogous in the peripheral compartment 2. A_3_ is the amount in brain tissue; V_e_ the volume fraction of brain ISF (mL/g); W_brain_ is brain weight; and C_3_ the concentration in brain tissue after correction for the intravascular content. Reprinted with permission from Reference [19]. Copyright 2022, Springer Nature.

The analysis of this type of model is based on the law of mass action for mass transfer between compartments, for which a series of corresponding differential equations can be formulated. To enable the fitting of model parameters to the data and to obtain values describing the rates and extent of tissue distribution, a compartmental model should not be over-parameterized, i.e., be only as complex as necessary. This may be illustrated by our recent approach to describe the PK behavior of the hydrophilic solutes presented above, [^13^C]sucrose and [^13^C]mannitol, using a three-compartment model. The model (Figure 4) consists of central and peripheral compartments, in addition to a brain compartment. The clearance rates across the BBB in either direction (CL_13_, Figure 4) are equal and based on passive diffusion for both the mannitol and sucrose, which are not substrates of known transporters and are metabolically stable in tissues. However, a PK model based only on symmetrical exchange across the BBB failed to adequately describe the time course in the brain. An additional term was required for clearance from the brain (CL_31_). This resulted in an extension of the two-compartment model used by Rapoport’s group for the description of the brain uptake of small nonelectrolytes [29]. The model (Figure 4) is expressed by the following mass transfer equations:

(8) $${\mathrm{dA}}_{1}/\mathrm{dt}=-{\mathrm{CL}}_{10}\cdot \frac{A_{1}}{V_{1}}-{\mathrm{CL}}_{12}\cdot \frac{A_{1}}{V_{1}}+{\mathrm{CL}}_{12}\cdot \frac{A_{2}}{V_{2}}-{\mathrm{CL}}_{13}\cdot \frac{A_{1}}{V_{1}}+\left(\left({\mathrm{CL}}_{13}+{\mathrm{CL}}_{31}\right)\cdot A_{3}\right))/\left(V_{e}\cdot W_{\mathrm{brain}}\right)$$

(9) $${\mathrm{dA}}_{2}/\mathrm{dt}={\mathrm{CL}}_{12}\cdot A_{1}/V_{1}-{\mathrm{CL}}_{12}\cdot A_{2}/V_{2}$$

(10) $${\mathrm{dA}}_{3}/\mathrm{dt}={\mathrm{CL}}_{13}\cdot A_{1}/V_{1}-\left(\left({\mathrm{CL}}_{13}+{\mathrm{CL}}_{31}\right)\cdot A_{3}\right))/\left(V_{e}\cdot W_{\mathrm{brain}}\right)$$

where,

C_1_ = A_1_/V_1_

C_2_ = A_2_/V_2_

C_3_ = A_3_/W_brain_

A_1_ and A_2_ denote the amount of analytes in the central and peripheral compartment, respectively. V_1_ and V_2_ are the volumes of these compartments. A_3_ is the amount in the brain compartment, V_e_ is equal to the volume of distribution of sucrose and mannitol in the brain tissue, expressed as a dimensionless volume fraction (assuming mL/g ≈ mL/mL). W_brain_ equals the brain weight. C_1_, C_2_, and C_3_ in Figure 4 are the concentrations. The clearance parameter CL_31_ denotes an efflux mechanism, which likely represents the bulk flow from the brain interstitial fluid, based on physiological considerations. The model parameters were fitted to the data by numerical solution of the differential equations using WinNonlin. Ve was fixed at 0.2, corresponding to the literature values of the extracellular volume fraction [30].

**Figure 5:**
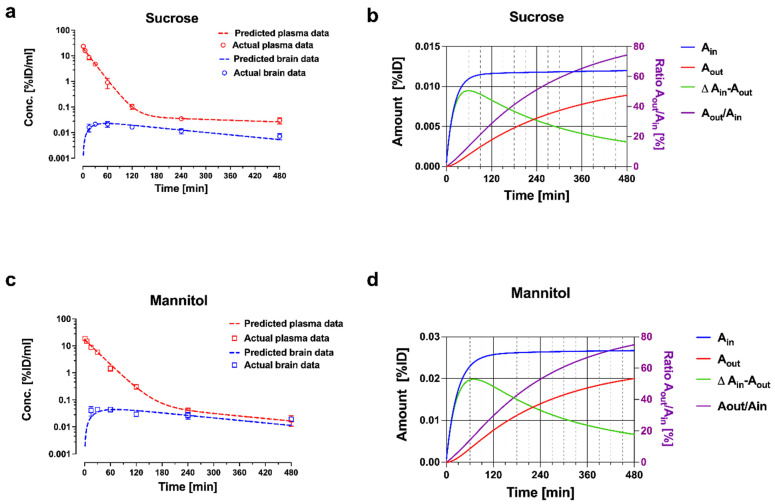
Plasma and brain pharmacokinetic data with model fitting for (**a**) sucrose and (**c**) mannitol. Plasma and brain concentrations are expressed in percent of injected dose (%ID/mL). Panels (**b**) and (**d**) depict cumulative amounts of sucrose and mannitol entering (A_in_) and leaving (A_out_) the brain compartment, and the ratio of A_out_/A_in_ over time. Reprinted with permission from Reference [19]. Copyright 2022, Springer Nature.

The estimates of the model parameters fitted to the data for mannitol and sucrose are listed in Table 1, and the plots of plasma and brain concentrations of the marker are shown in Figure 5. The parameter estimate for CL_13_ is the brain uptake clearance and is equivalent to K_in_. The fitted CL_13_ values for mannitol and sucrose (Table 1) are close to the K_in_ values obtained by the Patlak analysis over a 30 min period, and to the values of single time point analysis up to 30 min terminal sampling time (Figure 2 and Figure 3). The CL_13_ of mannitol is twice as high compared to sucrose (1.46 ± 0.02 µL·min^−1^·g^−1^ vs. 0.68 ± 0.005 µL·min^−1^·g^−1^). It is noteworthy that the CL_31_ estimates of both of the markers are not different (0.881 ± 0.20 µL·min^−1^·g^−1^ and 0.693 ± 0.106 µL·min^−1^·g^−1^). The same efflux value, despite differences in molecular weight, size, and octanol/water partition coefficient, is consistent with the bulk flow. This efflux clearance mechanism can be separated from diffusional exchange across the BBB because of the very low passive permeability of mannitol and sucrose. Therefore, the bulk flow clearance should have a significant impact on the brain kinetics of any endogenous substance or xenobiotic with similar physicochemical characteristics (low passive permeability, not a substrate of influx or efflux transport), and needs to be considered in the PK models used for analysis. The previous experimental estimates of the bulk flow in the brain typically relied on invasive techniques, such as ventriculo-cisternal perfusion or stereotaxic injection of tracers or dyes into the brain tissue [30,31,32]. The estimates of bulk flow in rodents range from 0.56 to 1.2 µL·min^−1^·g^−1^, as compiled in a recent review [33]. The above case study with sucrose and mannitol illustrates how relatively simple semi-physiological compartmental PK models can describe the PK behavior of extracellular markers, based on the measurement of whole tissue concentrations. The plots of plasma and brain concentration–time profiles of these markers after IV bolus injection (Figure 5) reveal that a model of BBB transport assuming unidirectional uptake cannot be applied for any time point beyond 30 min, because the brain concentrations already declined. This is consistent with the progressively decreasing *K_in_* estimates discussed above (Figure 2 and Figure 3).

More complex compartmental models have been described in the literature, beginning with the distributed model introduced by Fenstermacher, Patlak, and Blasberg [34] and later by Collins and Dedrick [35], which considers the exchange between the plasma compartment, the brain tissue, and the cerebrospinal fluid. Deeper insights into the PK characteristics of analytes can be gained when the experimental data are acquired from the additional tissue compartments. Of particular relevance in this regard is the in vivo microdialysis technique, which has been in use for drug analysis in brain interstitial fluid (ISF) since the 1990s [36,37,38]. Its value in brain uptake studies can be readily illustrated with the recent comparison of two isotopically labeled versions of sucrose [39]. A radiolabeled version, [^14^C]sucrose, accumulated in the whole brain tissue to about four-fold higher concentrations than the stable isotope labeled [^13^C]sucrose, while the brain ISF concentrations, measured in microdialysate from the striatum, were comparable. The discrepancy could be explained by the presence of a low amount of contaminants in the [^14^C]sucrose tracer solution [40], which are more lipophilic, more BBB permeable, and able to distribute into brain cells.

### Choice of Permeability Markers

While it is beyond the scope of this paper to broadly cover the range of markers used in the studies of the BBB, which have been discussed in several recent reviews [41,42], a comment is appropriate, considering the potential impact of technical issues on the results and interpretation of brain uptake studies. Evidently, any markers allowing only qualitative or semiquantitative analysis in blood and tissue cannot be reasonably used in PK models. The examples include horseradish peroxidase, (unlabeled) IgG, fibrinogen and dextrans, Trypan blue, and Evans blue [42]. Radiolabeled markers (e.g., radiolabeled forms of sucrose, mannitol, or inulin) can yield quantitative data, but careful chromatographic analysis of the integrity of the labeled substance in blood and tissue is required, as first pointed out decades ago [43], and illustrated in the preceding paragraph. Stable isotope-labeled and metabolically stable solutes, such as [^13^C] sucrose or [^13^C] mannitol, which lack affinity to transporter proteins appear as superior choices, because LC-MS/MS analysis is highly specific and sensitive [40,44], and the handling of radioisotopes is avoided. Fluorescein remains among the most frequently used permeability markers in the literature [42]. Typically, the total plasma and tissue concentrations are measured in plate readers, although it has been demonstrated that a seemingly higher brain uptake can be caused by altered plasma protein binding, especially under pathophysiological conditions, without actual changes in the BBB permeability [45]. Therefore, free fluorescein concentrations should be analyzed after ultrafiltration by sensitive chromatographic techniques. An additional caveat with fluorescein is that reports have implicated it as a substrate of probenecid sensitive efflux transporters for organic anions at the BBB [46,47]. Another drug initially considered to represent a hydrophilic marker suitable for PK studies of passive BBB permeability is S-atenolol, which was subsequently shown to be subject to efflux at the BBB [48].

## 4. Extent of Brain Drug Exposure

The majority of small molecule drugs will distribute to some degree into the cells or bind to the cell membranes, and this also applies to the brain tissue after passage of the BBB. The knowledge of the value of BBB uptake clearance alone is therefore insufficient. In addition, depending on the location of the drug target (e.g., membrane receptors vs. intracellular) the relevance of the total tissue concentrations is limited. Based on these considerations and on the free drug hypothesis, the group of Hammarlund-Udenaes developed a concept to describe the brain exposure, using a newly defined parameter, the unbound brain-to-plasma partition coefficient, *K_p,uu,brain_* [49,50]:

(11) $$K_{p,uu,brain}=\frac{AUC_{u,brainISF}}{AUC_{u,plasma}}$$

With *AUC_u,brainiSF_* denoting the AUC of the unbound concentrations in the brain interstitial fluid (ISF), and *AUC_u,plasma_* denoting the AUC of the free drug in the plasma. This parameter has been widely adopted, in particular in industrial CNS-drug development programs, and has largely supplanted the previously prevalent brain tissue to plasma partition coefficients, which represented the ratio between the total brain concentration and the total plasma concentration (*K_p_*) or the free plasma concentration (*K_p,u_*). *K_p,uu,brain_* is experimentally obtained from measurements of the brain extracellular fluid-drug concentrations by intracerebral microdialysis. The unbound plasma concentrations can also be determined by an indwelling microdialysis probe, or by ultrafiltration of the plasma samples ex vivo. As is evident from Equation (11), the determination of *K_p,uu,brain_* is independent of any specific compartmental model and can be performed after IV, bolus administration, or infusion. In practice, it is often based on a constant rate infusion schedule with a sufficiently long infusion time to achieve steady state conditions. At a steady state, the ratio of AUCs in Equation (11) can be replaced by the ratio of the free drug concentrations in the brain ISF and plasma. *K_p,uu,brain_* does not depend on partitioning processes inside the brain tissue between the ISF and cells. Further, because at steady state (*ss*) there is no net exchange between the drug in the brain ISF and plasma, the amounts, A, of drug influx into the brain and efflux from the brain are equal. With:

(12) $$\frac{dA_{in}}{dt}=CL_{in}\times C_{u,ss,plasma}=\frac{dA_{out}}{dt}=CL_{out}\times C_{u,ss,brainISF}$$

(13) $$K_{p,uu,brain}=\frac{C_{u,ss,brainISF}}{C_{u,ss,plasma}}=\frac{CL_{in}}{CL_{out}}$$

Therefore, *K_p,uu,brain_* can also be expressed as the ratio of the clearances *CL_in_*/*CL_out_*, where *CL_in_* comprises the sum of all of the passive and active influx clearances, and *CL_out_* represents the sum of all of the elimination clearances from the brain, i.e., passive and active BBB transport, metabolism in the brain, and bulk flow clearance [50]. If passive processes dominate the transport across the BBB, a value near unity would be expected. Correspondingly, if active influx is prevalent, *K_p,uu,brain_* is larger than unity, and if active efflux prevails, *K_p,uu,brain_* is less than one. This ratio provides, therefore, a powerful and simple tool to identify the principal transport mechanism of a drug at the BBB. It also follows from Equation (13) that the absolute clearance values cannot be assessed from this type of analysis. An estimate for *CL_in_* can be provided as complementary information from the measurement of the total brain concentrations, as discussed in the previous sections.

Another relevant parameter, the unbound drug volume of distribution, *V_u,brain_*, can be calculated from the measurement of microdialysate concentration and total brain tissue concentration at steady state:

(14) $$V_{u,brain}=\frac{A_{brain}}{C_{u,brainISF}}$$

where *A_brain_* is the amount in the brain per unit weight, after correction for the intravascular content. This parameter may also be estimated from an in vitro assay, the brain slice technique [51]. *V_u,brain_* provides information on the drug distribution inside the brain tissue between ISF and cells [50]. Importantly, *V_u,brain_*, according to Equation (14), is distinct from the apparent brain volume of distribution as used in the multiple time graphical analysis, which puts the plasma concentration in the denominator (see Equation (5)).

The synopsis of the plasma data, brain microdialysate sampling and whole tissue analysis provides unique pharmacokinetic insights, exemplified by a series of studies with morphine [52], its glucuronide metabolites [53,54,55,56], and the opioids, codeine and oxycodone [57,58]. The following conclusions can be derived from the comparison of these CNS-active drugs, as discussed before [50]: There is a vast range of BBB permeabilities, for example 167-fold in favor of oxycodone over morphine, and 1,150 fold over morphine-6-glucuronide. The *K_p_* and *K_p,u_* values also show differences of two log orders, because more lipophilic drugs tend to have a high affinity with brain tissue, which results in high values of *V_u,brain_*. In contrast, the *K_p,uu,brain_* values among these opioids differ only by a factor of about 10, between about 0.3 (morphine, morphine-6-glucuronide) and 3 (oxycodone). As explained above, these differences in *K_p,uu,brain_* can be attributed to the properties of the drugs as either a substrate of efflux transport (by P-gp and MRPs in case of morphine, morphine-6-glucuronide), of active uptake (oxycodone), or being a non-substrate (codeine) [52,56,57,58,59]. The impact of the transporters on the PK of opioids and its clinical implications have been discussed in a recent review [60]. Based on the relations outlined above, it can be argued that a complete picture of the delivery of CNS-active drugs requires knowledge of: (i) BBB permeability clearance; (ii) intra-brain distribution; and (iii) extent of equilibrium across the BBB. The appreciation of this concept is important in the decision processes for drug development.

## 5. Physiologically Based Pharmacokinetic Models

As a concept, PBPK dates back to the beginnings of the PK field [61,62]. Physiologically based models aim to describe the organism in terms of the compartments based on actual organs and their associated blood flow rates, with differential equations for mass transfer, tissue binding, and metabolic activity. In the classical compartmental models and semi-physiological models presented in the previous sections, all or part of the compartments were hypothetical spaces, defined by the apparent volumes of distribution, which were calculated from the plasma concentrations. While the compartmental and semi-physiologic models require fewer parameters, values for all or the majority of parameters can be obtained by fitting the models to experimental data (blood or plasma concentrations, and organ concentrations when available). A limitation of the compartmental models is that they cannot be readily scaled from one experimental species to another. In addition, the invasive nature of the animal experiments used in generating the input data for the models discussed above implies that a direct translation to the human condition is limited, which is one of the main drivers of the growing interest in PBPK models.

While there is no convincing evidence for major species’ differences in passive BBB permeability, the active transporters show marked differences among species. The recent advances in tandem mass spectrometry have enabled quantitative proteomics studies of transporter protein expression in animal and human brain microvessels [63]. Striking examples of species differences between a model organism and humans include the expression levels of the breast cancer-resistance protein, which is 1.85-fold higher expressed at the human BBB than in mice, and of p-glycoprotein (MDR), which is less expressed at the human BBB by a factor of 2.33 compared to mdr1a in mice. These quantitative data are now being utilized in the new field of pharmacoproteomics, to predict the K_p,uu,brain_ in humans from the data in experimental animals [64]. This will be beneficial in addressing the issue in PBPK involving the numerous parameters that these cannot be all determined by fitting to the available, sparse experimental data. Many of the parameters need to be fixed to the values from independent in vitro or in vivo studies, which are typically taken from the literature. The better estimates of transporter activity based on proteomics plugged into PBPK models would then facilitate interspecies scaling beyond the usage of the known values of organ weights, blood flow, and metabolic capacity, among others. The simulations with PBPK models have, in recent years, gained popularity as tools in drug development in industry and with regulatory agencies. The potential pitfalls in the widespread use of complex PBPK models due to over-parameterization and parameter optimization are under debate [65].

**Figure 6:**
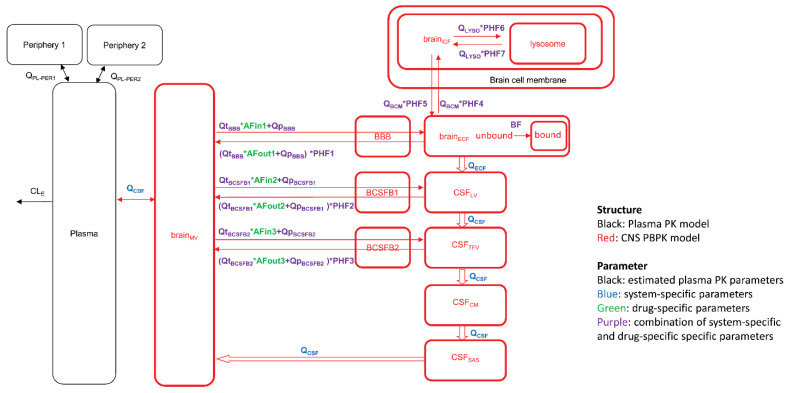
The model consists of a plasma pharmacokinetic (PK) model and a central nervous system (CNS) physiologically based pharmacokinetic (PBPK) model with estimated plasma PK parameters, and system-specific and drug-specific parameters (colors) for CNS. Peripheral compartments 1 and 2 are used when required to describe the plasma data adequately by a plasma PK model. AFin1–3, asymmetry factor into the CNS compartments 1–3; AFout1–3, asymmetry factor out from the CNS compartments 1–3; BBB, blood-brain barrier; BCSFB, blood-cerebrospinal fluid barrier; BF, binding factor; brainECF, brain extracellular fluid; brain_ICF_, brain intracellular fluid; brain_MV_, brain microvascular; CSF_CM_, cerebrospinal fluid in the cisterna magna; CSF_LV_, cerebrospinal fluid in the lateral ventricle; CSF_SAS_, cerebrospinal fluid in the subarachnoid space; CSF_TFV_, cerebrospinal fluid in the third and fourth ventricle; PHF1–7, pH-dependent factor 1–7; Q_BCM_, passive diffusion clearance at the brain cell membrane; Q_CBF_, cerebral blood flow; Q_CSF_, cerebrospinal fluid flow; Q_ECF_, brain_ECF_ flow; Q_LYSO_, passive diffusion clearance at the lysosomal membrane; Qp_BBB_, paracellular diffusion clearance at the BBB; Qp_BCSFB1_, paracellular diffusion clearance at the BCSFB1; Qp_BCSFB2_, paracellular diffusion clearance at the BCSFB2; Qt_BBB_, transcellular diffusion clearance at the BBB; Qt_BCSFB1_, transcellular diffusion clearance at the BCSFB1; Qt_BCSFB2_, transcellular diffusion clearance at the BCSFB2. From ref. [66] with permission under Creative Commons Attribution-NonCommercial-NoDerivs License.

In the BBB field, different academic groups proposed PBPK models aimed at predicting drug concentrations at the target sites, taking into account the different brain compartments, e.g., ISF and CSF, and eventually down to the level of intracellular vs. extracellular distribution and subcellular compartments, e.g., lysosomes [66,67,68]. Figure 6 depicts the scheme of such a model, which also includes different, connected CSF compartments, and asymmetry factors derived from the *K_p,uu_* values to account for the net effect of the influx and efflux transporters between compartments. An extensive list of published parameter values taken from experimental animals, human data, and in vitro studies has been compiled in a recent review [69]. Running simulations with this model for 10 diverse small molecule drugs with the published data on plasma kinetics, brain, and CSF concentrations, resulted in a less than two-fold prediction error of the concentration–time course in plasma, brain ISF, and two CSF sites (lateral ventricle and cisterna magna) [66].

The PBPK modeling is also increasingly applied to biotherapeutics, in particular monoclonal antibodies. An early example is the study of the distribution into tumor xenografts and organs of mice of an antibody targeting colon cancer [70]. Besides the specific target affinity, the later PBPK models also considered the role of the neonatal Fc receptor (FcRn) in the organ distribution and kinetics in plasma [71,72]. While the brain as a target organ had been previously ignored, in recent years the PBPK models have been proposed for the analysis of data generated with antibodies, which are under development as drug delivery vehicles, including antibodies targeted to the transferrin receptor (TfR) and insulin receptor [73,74,75]. The PBPK studies with TfR antibodies, in particular, benefit from a considerable body of published experimental data with full length antibodies, antibody fragments, bispecific antibodies, as well as antibody variants covering a wide range of target affinities, from low nanomolar to micromolar K_D_ values, over a range of doses. Two recent papers presented for the prediction of the brain disposition of TfR antibodies put an emphasis on the partially different sets of model parameters [73,75]. For example, in the model put forward by Pardridge and Chow, the binding kinetics and trafficking inside the BBB endothelial cells of the endogenous receptor ligand, transferrin, play major roles [75]. On the other hand, the model by Chang et al. did not consider transferrin-binding kinetics relevant with respect to the trafficking of TfR antibodies, but rather included the parameters for binding to FcRn and to TfR expressed on brain cells beyond the BBB [73]. Therefore, the output of simulations generated by these different models cannot be readily compared.

## 6. Conclusions and Perspectives

A selection of models is now at the disposal of investigators to evaluate pharmacokinetics and brain uptake in vivo in animal models and humans. The initial uptake rate measurements appear superficially straight forward, but they have associated caveats, which need to be considered. A full appreciation of the brain exposure of known compounds and of drug candidates requires knowledge of the uptake rate, the distribution in brain tissue, and the extent of equilibration across the BBB. The semi-physiological models, and complex PBPK models introduced in recent years, rely to a varying degree on the availability of the values for some of the parameters taken from independent in vitro or in vivo studies. The progress of quantitative pharmacoproteomics in experimental species and humans, which allows for interspecies adjustments and the scaling of the PK effect of transporter activities, as discussed in Section 5, is expected to enhance the predictive accuracy of the PBPK models. At this stage of maturation of the PK field, efforts should also be undertaken by the BBB PK expert community to push towards an improved quality of the host of studies being conducted in the neuroscience field, in which the measurement of the BBB permeability of drugs or markers serves only an ancillary purpose. In particular with respect to studies in disease models, the claims of altered permeability are often based on inadequate kinetic approaches, as pointed out for the use of markers viewed as imperfect by today’s standards (e.g., Evans Blue [42]), or neglecting the role of protein binding in plasma [45], or the common case of measurements at a single time point in brain tissue only, without considering the plasma kinetics as input.

## Figures and Tables

**Table 1:** Parameters of the 3 compartmental semi-physiologic model for fitting [^13^C_12_] sucrose and [^13^C_6_] mannitol plasma and brain uptake data simultaneously in adult male mice.

		[^13^C_12_] Sucrose			[^13^C_6_] Mannitol		
Parameters	Units	Value	SE	CV %	Value	SE	CV%
**V_1_**	mL	4.97 ^a^	0.326	6.56	6.06	0.34	5. 6
**V_2_**	mL	14.1 ^b^	8.28	58.6	3.22	0.63	19.7
**V_e_ (fixed)**	mL/g	0.2			0.2		
**CL_10_**	mL/min	0.226 ^c^	0.011	4.84	0.212	0.008	3.86
**CL_12_**	mL/min	0.019 ^d^	0.005	23.2	0.010	0.001	9.94
**CL_13_/Wbrain**	µL/(min × g)	0.068 ^e^	0.005	7.73	0.146	0.020	9.64
**CL_31_/Wbrain**	µL/(min × g)	0.693 ^f^	0.106	15.4	0.881	0.20	22.5
**W_brain_ (fixed)**	g	0.4			0.4		

Parameter definition as in Figure 4. Reprinted with permission from Reference [19]. Copyright 2022, Springer Nature. ^a^ *p* < 0.05 (*Z*-test value 2.31) compared to mannitol V_1_; ^b^ not significant (*Z*-test value 1.31) compared to mannitol V_2_; ^c^ not significant (*Z*-test value 1.03) compared to mannitol CL_10_; ^d^ not significant (*Z*-test value 1.77) compared to mannitol CL_12_; ^e^ *p* < 0.01 (*Z*-test value 3.85) compared to mannitol CL_13_/Wbrain; ^f^ not significant (*Z*-test value 0.8) compared to mannitol CL_31_/W_brain_.

## Data Availability

Not applicable.
